# Social-Ecological Predictors of Homophobic Name-Calling Perpetration and Victimization Among Early Adolescents

**DOI:** 10.1177/02724316211002271

**Published:** 2021-04-29

**Authors:** Alberto Valido, Gabriel J. Merrin, Dorothy L. Espelage, Luz E. Robinson, Kyle Nickodem, Katherine M. Ingram, America J. El Sheikh, Cagil Torgal, Javari Fairclough

**Affiliations:** 1 The University of North Carolina at Chapel Hill, USA; 2 Texas Tech University, Lubbock, USA; 3 University of Florida, Gainesville, USA

**Keywords:** homophobic name-calling, early adolescence, social ecological, longitudinal analysis

## Abstract

Bias-based aggression at school in the form of homophobic name-calling is quite prevalent among early adolescents. Homophobic name-calling is associated with low academic performance, higher risky sexual behaviors, and substance abuse, among other adverse outcomes. This longitudinal study examined risk and protective factors across multiple domains of the social ecology (individual, peer, family, school and community) and levels of analysis (within- and between-person) associated with homophobic name-calling perpetration and victimization. Students from four middle schools in the U.S. Midwest (*N* = 1,655; $\bar{X}$ age = 12.75; range = 10–16 years) were surveyed four times (Spring/Fall 2008, Spring/Fall 2009). For homophobic name-calling perpetration, significant risk factors included impulsivity, social dominance, traditional masculinity, family violence, and neighborhood violence; while empathy, peer support, school belonging, and adult support were significant protective factors. For homophobic name-calling victimization, significant risk factors included empathy (between-person), impulsivity, traditional masculinity, family violence, and neighborhood violence, while empathy (within-person), parental monitoring, peer support, school belonging, and adult support were significant protective factors.

## Introduction

Homophobic name-calling is a common form of bias-based aggression at school. Homophobic name-calling is understood as a form of gender-based harassment, consisting of pejorative labels (e.g., fag, homo) or denigrating phrases (e.g., don’t be a fag, that is so gay) aimed at youth perceived to be gay, lesbian, bisexual, queer, or gender nonconforming (Meyer, 2008). It is a manifestation of underlying homophobia or a pejorative expression of attitudes, beliefs, stereotypes, and behaviors against homosexuality (Wright, Adams, & Bernat, 1999). Although anyone can be targeted by homophobic name-calling, including youth who are not perceived to be a sexual or gender minority, gender and sexual minority youth report higher rates of victimization than heterosexual youth (Friedman et al., 2011; Williams, Connolly, Pepler, & Craig, 2005). In a recent survey of lesbian, gay, bisexual, transgender, and queer (LGBTQ) students’ experiences at school, more than 95% reported hearing words like “gay,” “faggot,” or “dyke” used in a negative way at school and about 70% reported being targets of verbal harassment themselves due to their sexual orientation (Kosciw, Greytak, Zongrone, Clark, & Truong, 2018). The same study found that 94% of LGBTQ students heard negative remarks about gender expression (not acting “masculine enough” or “feminine enough”), and 59.1% were verbally harassed because of their gender expression.

As a form of bias-based aggression, homophobic name-calling shares both similarities and differences with traditional bullying (Poteat & Espelage, 2005). While bullying may take the form of teasing, verbal epithets, and physical aggression, homophobic name-calling are targeted verbal attacks rooted in homophobia and prejudice and are more likely to be used when individuals are perceived to deviate from traditional gender norms and to assert dominance and power among peers (Birkett & Espelage, 2015; Poteat & Espelage, 2005). Fewer studies have focused on the outcomes associated with perpetration and victimization of homophobic name-calling, but research suggests that adolescents who engage in homophobic name-calling are also more likely to engage in bullying and vice versa (Birkett & Espelage, 2015; Merrin et al., 2018). As such, homophobic name-calling is a distinct phenomenon that may or may not share associations uncovered in the bullying literature, warranting further examination of the phenomenon in the present study.

Being the target of homophobic name-calling is often associated with poor mental health symptoms, such as higher incidence of anxiety, depression, and suicide-related behaviors among adolescents (Almeida, Johnson, Corliss, Molnar, & Azrael, 2009; D’Augelli, 2002; Espelage, Aragon, Birkett, & Koenig, 2008; Poteat & Espelage, 2007; Rivers, 2001). It has also shown associations with increased absenteeism, lower academic performance, and higher incidence of risky sexual behaviors and substance abuse, among other negative outcomes (Biddulph, 2008; Espelage et al., 2008; Rivers, 2000, 2004). Due to the negative consequences associated with homophobic name-calling victimization, previous studies have called for longitudinal analyses that go beyond identifying individual risk factors to include both risk and protective factors across multiple domains of the social-ecological context during adolescence (Basile, Espelage, Rivers, McMahon, & Simon, 2009; Espelage & Swearer, 2010; Hong & Garbarino, 2012).

### Theoretical Framework

The social-ecological correlates of homophobic name-calling can be found among peers, family, the school, and the community, which are all embedded within the broader society and can influence homophobic attitudes and behaviors at school (Espelage & Swearer, 2010; Hong & Garbarino, 2012; Kosciw, Greytak, & Diaz, 2009). The social-ecological framework is rooted in the theoretical work of Urie Bronfenbrenner (1979), who proposed that human development and behavior are a product of interactive interplay between the individual and multiple layers of one’s social environment. The present study is informed by the extant literature on homophobic name-calling and peer victimization at different domains of the social ecology. Not all domains of Bronfenbrenner’s theory were included in this study. Based on the data available and the extant literature, only the factors the authors considered most relevant for homophobic name-calling were included. In addition, the present study is guided by the theoretical framework of risk and protective factors, which have been identified as potential targets for violence and aggression prevention and are defined as factors that may increase or lessen the incidence of aggressive behaviors (Centers for Disease Control and Prevention, 2020; Gómez-Leal, Megías-Robles, Gutiérrez-Cobo, Cabello, & Fernández-Berrocal, 2020; Hong & Garbarino, 2012; Lösel & Farrington, 2012). The purpose of the present study is to examine risk and protective factors within and across social-ecological domains that contribute to longitudinal patterns of homophobic name-calling perpetration and victimization across early adolescence. Next, we discuss each domain and specific associations for victimization and perpetration of homophobic name-calling. For some associations, the authors do not have specific hypotheses (e.g., empathy and homophobic victimization [HV]); however, we will be examining all predictors for both homophobic name-calling victimization and perpetration.

### Individual Domain Associations With Homophobic Name-Calling

Individual domain characteristics associated with homophobic name-calling are gender, age, traditional masculinity attitudes, social dominance orientation, empathy, and impulsivity (Poteat & DiGiovanni, 2010). Studies have documented pronounced differences between boys’ and girls’ engagement in homophobic name-calling (Espelage, Basile, Leemis, Hipp, & Davis, 2018; Poteat & Espelage, 2005; Poteat, O’Dwyer, & Mereish, 2012), with boys consistently reporting higher occurrences of both perpetration and victimization. Researchers argue that distinct gender socialization processes account for behavioral discrepancies (Phoenix, Frosh, & Pattman, 2003; Poteat et al., 2012). Heteronormative gender stereotypes foster homophobic attitudes among boys and girls who are socialized to adopt a traditionally masculine ideology (Pleck, Sonenstein, & Ku, 1994). Boys may be more likely to engage in homophobic name-calling or perform heterosexual acts to assert their masculinity (Korobov, 2004; Pascoe, 2011; Poteat et al., 2012). In addition, age-related trends have been identified in the literature with younger adolescents having a higher endorsement of homophobia and prejudice which could increase homophobic name-calling perpetration and victimization among early adolescents (Poteat & Russell, 2013; Robinson, Espelage, & Rivers, 2013). Findings also suggest that homophobic name-calling victimization may decrease among older adolescents reaching a peak in middle school where students may have the highest levels of prejudice (Birkett, Newcomb, & Mustanski, 2015).

Similarly, studies have found that homophobic name-calling perpetration serves to establish and maintain social dominance hierarchies within and between adolescent peer groups through the promotion of heteronormative gender roles (Birkett & Espelage, 2015; Pellegrini & Long, 2002; Poteat & DiGiovanni, 2010). Social dominance has been defined as attitudes or behaviors intended to establish or perpetuate hierarchies of power, status, or control over resources (Hawley, 1999). The use of homophobic name-calling within the context of social dominance may put the targeted student in a subordinate position due to the stigma associated with identifying as a sexual or gender minority (Poteat & DiGiovanni, 2010).

Furthermore, an individual’s level of empathy and impulsivity may play a role in homophobic name-calling (Espelage et al., 2019; Poteat & Espelage, 2005; Zych, Ttofi, & Farrington, 2019). Students with low levels of empathy may lack the ability to understand and identify with the perspective of others, which could be associated with higher engagement in homophobic name-calling perpetration (Poteat, DiGiovanni, & Scheer, 2013). For example, Poteat and colleagues (2013) found that empathy predicted a lower incidence of prejudice and bullying behaviors, which in turn was associated with lower homophobic name-calling among 581 high school students. Similarly, impulsivity has been associated with higher engagement in multiple forms of aggression, including homophobic name-calling (Merrin et al., 2018). In this longitudinal study of middle school students’ (*N* = 190) social networks, impulsivity was strongly associated with higher engagement in homophobic name-calling over time (Merrin et al., 2018). Perhaps these students lack the regulatory abilities to pre-emptively imagine the consequences of their actions in contrast to following suit of others or gaining social standing.

### Family Domain Associations With Homophobic Name-Calling

Family domain characteristics and interaction patterns influence adolescents’ social, emotional, and cognitive development. Numerous studies have found that exposure to family violence, parental monitoring, and family social support often predict bullying involvement (victimization and perpetration) (Bowes, Maughan, Caspi, Moffitt, & Arseneault, 2010; Espelage, Bosworth, & Simon, 2000; Low & Espelage, 2013). Both direct exposure and indirect exposure to family violence have been associated with bullying victimization and perpetration (Espelage et al., 2000; Voisin & Hong, 2012). Furthermore, children who are victims of bullying often experience abuse in the home or inconsistent parenting (Espelage, Low, & De La Rue, 2012; Georgiou & Fanti, 2010).

Parental monitoring and surveillance have been found to influence adolescents’ involvement in bullying and other forms of youth aggression. Studies found bullying and other aggressive behavior tends to occur when parents are absent or when youth are not provided sufficient supervision (Doty, Lynne, Metz, Yourell, & Espelage, 2020; Espelage et al., 2000; Georgiou & Fanti, 2010; Low & Espelage, 2013). Within the family domain, consistent but not excessive parental monitoring has been identified as a protective factor for bullying and involvement in types of aggression (victimization and perpetration) among adolescents (Doty et al., 2020; Espelage, 2014; Li, Stanton, & Feigelman, 2000).

Family social support is the level of recognition, approval, and care that youth receive from family members and has been correlated with bullying and other types of aggression involvement. Experiencing parental rejection and inadequate support from family members has been associated with bullying perpetration (Georgiou, 2008). Other studies have found that supportive family relationships can mitigate the impact of bullying involvement among adolescents (Bowes et al., 2010; Holt & Espelage, 2007). However, fewer studies have focused on family social support as it relates to protection against being a target of homophobic name-calling (Espelage et al., 2019). One exception was a cross-sectional study of 15,923 middle and high school students in the Midwest United States which found that high levels of parental support were correlated with lower levels of homophobic name-calling victimization for both White and racial minority students (Poteat, Mereish, DiGiovanni, & Koenig, 2011). Thus, family support may be protective of both homophobic name-calling victimization and perpetration.

A recent review of protective factors associated with homophobic name-calling and its consequences highlighted that few individual and family characteristics have been examined (Espelage et al., 2019). Specifically, studies examining the role of family support in homophobic name-calling have only studied parental support and neglected to study the role of other family members. The current study measures family support broadly to capture all types of family support. In addition, the majority of studies on protection against homophobic name-calling have focused on the peer- and school-domain (Espelage et al., 2019). To date, there are no longitudinal studies on whether family violence, parental monitoring, and family social support protect against homophobic name-calling involvement.

### Peer Domain Associations With Homophobic Name-Calling

Relying on close non-family others such as friends, romantic partners, or individuals considered “found family” or “chosen family” for support is a common experience among LGBTQ youth (McConnell, Birkett, & Mustanski, 2015). Furthermore, in a sample of LGBTQ participants, Blair and Pukall (2015) found that compared with heterosexual individuals, individuals interested in same-sex relationships were more likely to place more value on friends’ or chosen family’s approval of a significant other than their own family. In addition, Hailey, Burton, and Arscott (2020) found in a synthesis of studies on chosen families among African American LGBTQ youth that having a support network of other African American LGBTQ individuals was important for healing psychological distress (with regard to racial trauma as well), practicing coping skills, and developing an identity. This was especially true for individuals who experienced family rejection. Due to the protective nature of peer social support on minority students, having social support from friends may also have a protective effect on homophobic name-calling perpetration and victimization.

Peer social support could also play a protective role against homophobic name-calling victimization among heterosexual youth by providing support for youth being targeted by perpetrators. However, peer networks have previously been identified as a context that promotes homophobic name-calling perpetration among adolescents (Birkett & Espelage, 2015). The homophily hypothesis posits that youth tend to become friends with peers who share similar characteristics as themselves (Kandel, 1978). According to this theory, the peer group acts as a medium for the socialization of prejudice and homophobia which contribute to engagement in homophobic name-calling perpetration (Poteat, 2008). Therefore, peer social support may have a dual role both helping to reinforce prejudice and homophobia among perpetrators and potentially buffering against victimization.

### School Domain Associations With Homophobic Name-Calling

School belonging is commonly described as students’ sense of being accepted, valued, included, and encouraged by peers and other school community members (Goodenow, 1993). Feelings of school belonging can be facilitated or inhibited by characteristics of school climate. A study of 1,415 students in 28 high schools found that experiences of peer victimization can diminish feelings of school safety for gender nonconforming youth; however, a positive school climate can foster feelings of school safety for gender nonconforming youth (Toomey, McGuire, & Russell, 2012). Research shows that school belonging can protect against homophobic name-calling (Espelage et al., 2019) and may foster climates where homophobic name-calling is not tolerated (Hong & Garbarino, 2012).

In addition, social support from trusted non-family adults has been identified as a robust protective moderating element in the pathway from victimization to mental health issues among LGBTQ youth cross-culturally (Antonio & Moleiro, 2015). For transgender and gender variant youth specifically, a recent review synthesized several qualitative and quantitative studies that described the globally positive impact of having a trusted adult at school (Johns, Beltran, Armstrong, Jayne, & Barrios, 2018). Specifically, they note that youth report that having someone to help them navigate school systems and advocate for them at school makes them attend school more often and achieve better academic outcomes (Johns et at., 2018). The role of supportive adults at school is equally important for youth regardless of their sexual orientation or gender identity and may be associated with both lower perpetration and victimization of homophobic name-calling.

### Neighborhood/Community Domain Associations With Homophobic Name-Calling

School climate is heavily impacted by the larger community in which schools are embedded (Espelage, 2014; Kitsantas, Ware, & Martinez-Arias, 2004; Kosciw et al., 2009). Exposure to community violence has been broadly defined as youth’s encountering incidences of violence in their neighborhood or community (Laursen, 2011). Exposure to community violence has been identified as a risk factor that is associated with numerous negative developmental outcomes for youth such as externalizing behaviors (Barkin, Kreiter, & DuRant, 2001), bullying perpetration and victimization (Low & Espelage, 2014). Forber-Pratt and Espelage (2018) suggest that due to the heteronormative nature of gangs that value hypermasculinity, youth who are exposed to gangs in their neighborhood may report greater engagement in homophobic name-calling at school.

### The Current Study

The extant literature reveals a combination of risk and protective factors associated with homophobic name-calling at multiple domains of the social-ecological model. However, most studies have employed cross-sectional designs, which can only inform between-person differences, but not variation within a person over time. Furthermore, the few longitudinal studies on homophobic name-calling among adolescents have yet to include within-person variability, thus confounding within- and between-person sources of variability. Also, no study to date has simultaneously examined a comprehensive set of social-ecological domains with a risk and protective factors framework (Espelage et al., 2019; Orue & Calvete, 2018; Poteat et al., 2013). To address these gaps, the present study employs a longitudinal multilevel design that disentangles within- and between-person variability in both homophobic name-calling perpetration and victimization while also accounting for similar longitudinal processes at the individual, family, peer, school, and neighborhood domains.

While between-person variation informs overall homophobic name-calling trends, examining within-person variation is critical to understanding how time-specific contextual factors in the social-ecological model are associated with concurrent levels of homophobic name-calling. To illustrate the interpretation and importance of this statistical technique, consider the findings from Merrin, Davis, Berry, and Espelage (2019) relating parental monitoring to deviant and violent behavior from 5th to 11th grade. Youth who experienced high parental monitoring, on average, reported lower rates of deviant and violent behaviors (between-person association). When parental monitoring at a given time point was higher than typical for a youth, this resulted in additional reduction of deviant and violent behaviors (within-person association) The study also found a significant interaction between within-person parental monitoring and peer deviance, for youth who typically experienced high parental monitoring and low peer deviance, instances with additionally heightened parental monitoring resulted in increased deviant and violent behaviors, possibly due to youth interpreting the increased monitoring as unnecessarily intrusive. If only one level of this association is examined, we may miss nuance in the relationships between variables that vary not only between-person but also within-person (Curran & Bauer, 2011). Because the levels carry different substantive meanings, they also have the potential to differ in the magnitude and direction of the effects and should be examined separately. In addition, by examining a sample of early adolescents, the current study shines a light on socio-ecological processes that can be targets of early prevention programming aimed at reducing homophobic name-calling.

We hypothesize that the following protective factors will be significantly associated with a lower incidence of homophobic name-calling perpetration and victimization at both the within- and between-person levels of analysis: (H1) individual domain empathy; (H2) family social support; (H3) parental monitoring; (H4) school belonging; (H5) social support from adults at school; and (H6) social support from peers. In addition, we hypothesize that the following risk factors will be associated with higher homophobic name-calling perpetration and victimization at both the within- and between-person levels of analysis: (H7) individual domain impulsivity; (H8) family violence; and (H9) neighborhood violence. Finally, we hypothesize significant associations between the following risk factors and higher homophobic name-calling perpetration (not victimization): (H10) social dominance; and (H11) traditional masculinity.

## Method

### Participants and Procedures

Students from four middle schools in the Midwest (*N* = 1655) were surveyed four times (Spring/Fall 2008; Spring/Fall 2009). Data were collected every semester in middle school to capture temporal associations among risk and protective factors of multiple forms of violence. New students were enrolled at each wave, and students may not have been surveyed at some waves because of moving to another school or being chronically absent. Students in this data set had to have data at Wave 1. Response rates ranged from 92% to 95% at each wave. At baseline, students were in fifth (4.0%), sixth (34.1%), seventh (34.2%), and eighth (27.7%) grade. Participants’ average age was 12.75 years (range 10–16), 49.5% were female, 47.9% African American, 36.1% White, 3.3% Hispanic, 1.6% Asian/Pacific Islander, and 10.8% other (Table 1). Free/reduced-price lunch rates ranged from 60% to 73% across the schools. A total of 990 (93.2%) students identified as straight/heterosexual, 0 (0%) identified as gay/lesbian, 5 (0.5%) identified as bisexual, and 64 (6.3%) indicated they were unsure of their sexual orientation at Wave 1.

**Table 1.** Descriptive Statistics, Means, and Standard Deviations of All Variables Across Time.

*Variables at each level*	Wave 1		Wave 2		Wave 3		Wave 4		Range
	*$\bar{X}$/n*	*SD/*%	*$\bar{X}$/n*	*SD/*%	*$\bar{X}$/n*	*SD/*%	*$\bar{X}$/n*	*SD/*%	
Demographics (*N* = 1,655)									
Age	12.75	1.07							10–16
Male	836	50.5%							
Female	819	49.5%							
Asian/Pacific Islander	27	1.6%							
African American	793	47.9%							
Hispanic	55	3.3%							
White	597	36.1%							
Other (race/ethnicity)	179	10.8%							
Heterosexual	990	93.2%							
Bisexual	5	0.5%							
Not sure (sexual orientation)	64	6.3%							
Parent education (highest)									
High school	391	26.5%							
College	727	49.4%							
Graduate school	355	24.1%							
Homophobic name-calling									
Homophobic perpetration	0.70	0.95	0.81	0.96	0.89	1.05	0.84	1.06	0–4
Homophobic victimization	0.38	0.64	0.50	0.85	0.48	0.80	0.46	0.81	0–4
Individual level									
Empathy	1.76	0.88	1.83	0.82	1.79	0.85	1.79	0.87	0–4
Impulsivity	1.35	0.99	1.29	1.00	1.26	1.04	1.25	1.06	0–4
Social dominance	1.51	0.96	1.57	1.00	1.54	1.00	1.56	1.08	0–4
Traditional masculinity	0.92	0.50	0.90	0.56	0.89	0.58	0.94	0.63	0–3
Family level									
Family violence	1.38	1.24	1.33	1.29	1.27	1.34	1.12	1.18	0–5
Parental monitoring	2.34	0.72	2.27	0.83	2.18	0.86	2.21	0.85	0–3
Family social support	1.63	0.50	1.59	0.51	1.56	0.56	1.58	0.51	0–2
School and neighborhood level									
Neighborhood violence	1.19	1.00	1.21	1.00	1.20	0.99	1.07	1.00	0–3
School belonging	1.91	0.60	1.94	0.64	1.91	0.66	1.97	0.62	0–3
Social support from adults at school	1.13	0.56	1.18	0.56	1.12	0.56	1.22	0.55	0–2
Peers level									
Social support from peers	1.28	0.58	1.31	0.59	1.28	0.57	1.23	0.58	0–2

All students in the participating schools were recruited for the study. A waiver of parental consent was approved by the institutional review board and school districts, so parents only returned signed consent forms if they did not wish for their child to participate. During survey administrations, trained proctors described the study, collected student assent, and read the survey aloud while students completed it. Student assent was obtained at each of the subsequent follow-up waves prior to the start of the survey.

### Measures

Participants completed demographic questions on *sex* (male/female), *age* (open-ended), *grade, race/ethnicity* (Black, Asian, Pacific Islanders, White, Hispanic, Native American), and highest *parental education* (less than high school, high school or GED, some college, graduated from college, some graduate school, and graduate or professional [e.g., accountant, doctor]), and sexual orientation (heterosexual/straight, gay/lesbian, bisexual, or not sure; assessed only at Wave 1). Full descriptions of measures are available in Supplemental Table 1.

### Outcome Measures

#### Homophobic name-calling perpetration and victimization

The 10-item Homophobic Content Agent Target Scale was used to assess homophobic name-calling perpetration and victimization (Poteat & Espelage, 2005, 2007). Across the four waves, internal consistency reliability as measured by omega (ω) ranged from .88 to .90 for perpetration and from .86 to .89 for victimization.

### Risk and Protective Factors

Predictors were measured at 4 time points (Wave 1–Wave 4) and included participants’ perceptions of individual, family, school, neighborhood, and peer domain factors. *Perceptions of individual domain factors* that were assessed: (a) impulsivity, four items, ωs = .76 to .81 (Bosworth, Espelage, & Simon, 1999); (b) empathy, five items, ωs = .72 to .80 (Bosworth & Espelage, 1995); (c) traditional masculinity, seven items, ωs = .81 to .86 (Chu, Porche, & Tolman, 2005); and (d) social dominance, seven items, ωs = .83 to .87 (Pellegrini & Long, 2002). *Perceptions of family domain factors* that were assessed: (a) family conflict and hostility, three items, ωs = .81 to .83 (Thornberry, Krohn, Lizotte, Tobin, & Smith, 2003); (b) parental monitoring, eight items, ωs = .90 to .92 (Arthur, Hawkins, Pollard, Catalano, & Baglioni, 2002); and (c) family social support, three items, ωs = .79 to .85 (Vaux, 1988). *Perceptions of school and neighborhood domain factors* included: (a) exposure to community violence, five items, ωs = .92 across all waves (Low & Espelage, 2014); (b) social support from adults at school, three items, ωs = .80 to .84 (Vaux, 1988); and (c) school sense of belonging, four items, ωs = .66 to .76 (Goodenow, 1993). *Perceptions of peer domain factors* included peer social support, three items, ωs = .84 to .87 (Vaux, 1988; Vaux et al., 1986).

### Analytic Plan

A longitudinal latent growth model was used to examine trajectories of homophobic name-calling perpetration and homophobic name-calling victimization among middle school students across four waves of data conditioned on both time-variant (within-person) and time-invariant (between-person) predictors. The intraclass correlations for homophobic perpetration (HP) was .52, indicating that 52% of the total variation reported perpetration was variation between students, whereas 48% was variation within a student across time. Similarly, the intraclass correlation for HV was .59. With a substantial proportion of the total variation attributable to both within- and between-person components, explaining the variation in the outcomes necessitates including in the model both within-person variables that vary across time for the same individual and between-persons variables that are stable across time for a given individual and highlight differences between persons. To do so, we fit a taxonomy of unconditional and conditional growth models. First, an unconditional model (Model 1) established plausible rates of change for HP and HV. The model included random linear and quadratic slopes, meaning a separate linear and quadratic slope was estimated for each student.

We then tested our hypotheses by examining groups of conditional growth models for each social ecological domain (individual, family, peer, school, neighborhood). A separate model for each domain was fit (Models 2–7) followed by a final competing model that included predictors across all social domains (Model 8).

The within-person predictors were person-mean centered to examine how individuals deviate from their own “typical” levels (within-person average). The between-person predictors were grand-mean centered to examine average differences between people. The centering strategies separate within- and between-person variance, thereby making within- and between-person representations of the predictors orthogonal to each other (Wang & Maxwell, 2015). As such, within-person predictors treat individuals as their own control, thereby adjusting for all observed and unobserved between-person confounds. All models were run using Mplus 8.4 using the robust maximum likelihood estimator, which adjusts for potential non-normality in the data by estimating robust standard errors using a Huber-White sandwich estimator. Full information maximum likelihood was used to address missing data. Baseline (Wave 1) self-reports of biological sex, race/ethnicity, highest parental education, and age were used as covariates in all conditional analyses (Models 2–8). Models were run separately and then in a combined model for two important reasons: to be comprehensive in our model building approach and to show which domains of the social-ecological context could overpower others or conversely whether the domains are independent from each other. By doing so, we offer a more nuanced and comprehensive view of the social-ecological model that allows us to examine all domains independently of each other and competing with each other to explain the etiology of homophobic name-calling perpetration and victimization.

## Results

### Descriptive Statistics

Descriptive statistics across time are presented in Table 1. Correlations among study variables are presented in Supplemental Table 2.

### Homophobic Perpetration

#### Individual

Table 2 (Model 3) presents the within- and between-person predictors for the individual domain. Females reported lower rates, and older individuals reported slightly higher rates of HP over time, and African Americans reported higher rates of HP compared with their white counterparts. At the within-person level, at time points when individuals reported higher rates of empathy compared with their “typical” levels (an individual’s average level over time), they, in turn, reported lower rates of HP at the same time point. Conversely, at time points when individuals reported higher rates of impulsivity and dominance compared with their “typical” levels, they, in turn, reported higher rates of HP at the same time point. At the between-person level, on average, individuals who reported higher average rates of empathy compared with other people who reported lower rates of HP across time. Individuals who reported higher average rates of impulsivity, dominance, and traditional masculinity reported higher rates of HP across time.

**Table 2.** Estimates of Fixed and Random Effects From a Series of Growth Models Predicting Homophobic Perpetration.

Parameter estimates (*SE*)								
*Variables at each level*	Model 1	Model 2	Model 3	Model 4	Model 5	Model 6	Model 7	Model 8
Fixed effects								
Intercept	0.75*** (0.024)	0.72*** (0.052)	0.70*** (0.045)	0.81*** (0.049)	0.71*** (0.052)	0.75*** (0.051)	0.82*** (0.052)	0.79*** (0.045)
Linear slope	0.12*** (0.027)	0.12*** (0.027)	0.13*** (0.027)	0.11*** (0.027)	0.12*** (0.027)	0.12*** (0.027)	0.13*** (0.026)	0.13*** (0.027)
Quadratic slope	−0.03*** (0.008)	−0.03*** (0.008)	−0.03*** (0.009)	−0.03*** (0.008)	−0.03*** (0.008)	−0.03*** (0.008)	−0.03*** (0.008)	−0.03*** (0.008)
Demographics								
Female		−0.22*** (0.038)	−0.07 (0.034)	−0.27*** (0.034)	−0.19*** (0.040)	−0.21*** (0.036)	−0.20*** (0.036)	−0.17*** (0.033)
Parent education: College educated		−0.02 (0.045)	0.002 (0.038)	−0.05 (0.040)	−0.01 (0.045)	−0.01 (0.043)	0.02 (0.043)	−0.02 (0.037)
Parent education: Graduate school		−0.05 (0.052)	−0.04 (0.046)	−0.05 (0.047)	−0.03 (0.053)	0.01 (0.050)	0.01 (0.05)	−0.04 (0.044)
Age		0.04* (0.018)	0.02 (0.016)	0.01 (0.016)	0.04* (0.018)	0.02 (0.017)	0.01 (0.017)	0.003 (0.015)
African American		0.30*** (0.042)	0.16*** (0.038)	0.20*** (0.038)	0.28*** (0.043)	0.19*** (0.041)	0.02 (0.047)	0.09* (0.041)
Other		0.08 (0.055)	0.04 (0.048)	0.05 (0.049)	0.07 (0.054)	0.04 (0.052)	−0.05 (0.054)	0.03 (0.047)
Individual level								
WP empathy			−0.08*** (0.014)					−0.05*** (0.014)
WP impulsivity			0.10*** (0.015)					0.08*** (0.015)
WP dominance			0.13*** (0.015)					0.11*** (0.014)
WP traditional masculinity			0.01 (0.024)					−0.01 (0.024)
BP empathy			−0.15*** (0.029)					−0.12*** (0.030)
BP impulsivity			0.36*** (0.024)					0.24*** (0.026)
BP dominance			0.21*** (0.022)					0.16*** (0.022)
BP traditional masculinity			0.30*** (0.049)					0.15** (0.046)
Family level								
WP family violence				0.11*** (0.014)				0.07*** (0.014)
WP parental monitoring				−0.09*** (0.020)				−0.08*** (0.020)
WP family support				−0.02 (0.030)				0.04 (0.031)
BP family violence				0.32*** (0.022)				0.19*** (0.022)
BP parental monitoring				−0.25*** (0.035)				−0.16*** (0.033)
BP family support				0.22*** (0.058)				0.34*** (0.057)
Peer level								
WP peer support					−0.14*** (0.024)			−0.09*** (0.026)
BP peer support					−0.09 (0.048)			0.11* (0.050)
School level								
WP school belonging						−0.11*** (0.024)		−0.08** (0.023)
WP adult support						−0.08** (0.025)		−0.04 (0.026)
BP school belonging						−0.49*** (0.057)		−0.16** (0.050)
BP adult support						−0.12* (0.055)		−0.18** (0.054)
Neighborhood level								
WP neighborhood violence							0.15*** (0.019)	0.08*** (0.019)
BP neighborhood violence							0.31*** (0.028)	0.05 (0.025)
Random effects								
WP intercept	0.28*** (0.014)	0.28*** (0.014)	0.28*** (0.014)	0.27*** (0.013)	0.28*** (0.014)	0.28*** (0.014)	0.28*** (0.014)	0.27*** (0.013)
BP intercept	0.70*** (0.045)	0.67*** (0.044)	0.54*** (0.038)	0.62*** (0.041)	0.68*** (0.044)	0.62*** (0.041)	0.63*** (0.041)	0.51*** (0.036)
Linear Slope	0.49*** (0.064)	0.49*** (0.064)	0.48*** (0.063)	0.48*** (0.063)	0.48*** (0.063)	0.49*** (0.064)	0.45*** (0.062)	0.45*** (0.061)
Quadratic slope	0.04*** (0.006)	0.04*** (0.006)	0.04*** (0.006)	0.04*** (0.006)	0.04*** (0.006)	0.04*** (0.006)	0.04*** (0.006)	0.04*** (0.006)
Fit indices								
−2 Log-likelihood	16,331.684	16,233.248	15,500.942	15,716.012	16,187.594	16,002.426	16,008.882	15,150.426
AIC	16,351.684	16,265.247	15,548.942	15,760.013	16,223.594	16,042.426	16,044.881	15,226.426
BIC	16,419.662	16,374.013	15,712.091	15,909.565	16,345.955	16,178.383	16,167.243	15,484.744
Degrees of freedom	10	16	24	22	18	20	18	38

*Note.* Male is the reference category for gender and White is the reference category for race. Model 1 is a conditional growth model with linear and quadratic effects of growth that allows growth to vary randomly between people. Model 2 added demographic variables. Model 3 includes individual-level variables. Model 4 includes family-level variables. Model 5 includes peer-level variables. Model 6 includes school-level variables. Model 7 includes neighborhood-level variables. Model 8 includes variables across all social ecological domains. Intercept, random linear slope, and quadratic slope were allowed to co-vary. Covariances are not shown for ease of reading. WP = within-person; BP = between-person; SES = socioeconomic status; AIC = Akaike information criterion; BIC = Bayesian information criterion.

* *p* < .05. **p < .01. ***p < .001.

#### Family

Table 2 (Model 4) presents the within- and between-person predictors for the family domain. At the within-person level, at time points when individuals reported higher rates of family violence compared with their “typical” levels, they, in turn, reported higher rates of HP at the same time point. Conversely, at time points when individuals reported higher rates of parental monitoring compared with their “typical” levels, they, in turn, reported lower rates of HP at the same time point. At the between-person level, on average, individuals who reported higher average rates of family violence and family support reported higher rates of HP across time. Individuals who reported higher average rates of parental monitoring reported lower rates of HP across time.

#### Peer

Table 2 (Model 5) presents the within- and between-person predictors for the peer domain. At the within-person level, at time points when individuals reported higher rates of peer support compared with their “typical” levels, they, in turn, reported lower rates of HP at the same time point. At the between-person level, rates of peer support were not significantly associated with HP across time.

#### School

Table 2 (Model 6) presents the within- and between-person predictors for the school domain. At the within-person level, at time points when individuals reported higher rates of school belonging and adult support compared with their “typical” levels, they, in turn, reported lower rates of HP at the same time point. At the between-person level, on average, individuals who reported higher average rates of school belonging and adult support reported lower rates of HP across time.

#### Neighborhood

Table 2 (Model 7) presents the within- and between-person predictors for the neighborhood domain. At the within-person level, at time points when individuals reported higher rates of neighborhood violence compared with their “typical” levels, they, in turn, reported higher rates of HP at the same time point. At the between-person level, on average, individuals who reported higher average rates of neighborhood violence reported higher rates of HP across time.

#### Competing model

Table 2 (Model 8) presents the within- and between-person predictors for the competing model. As a final step, we included predictors across all social domains in one competing model to examine key predictors while controlling for all other variables. At the within-person level, at time points when individuals reported higher rates of empathy, parental monitoring, peer support, and school belonging, compared with their “typical” levels, they, in turn, reported lower rates of HP at the same time point. Conversely, at time points when individuals reported higher rates of impulsivity, dominance, family violence, and neighborhood violence compared with their “typical” levels, they, in turn, reported higher rates of HP at the same time point. At the between-person level, individuals who reported higher average rates of impulsivity, dominance, traditional masculinity, family violence, and family support, they reported higher rates of HP across time. Individuals who reported higher average rates of empathy, parental monitoring, school belonging, and adult support reported lower rates of HP across time.

### Homophobic Victimization

#### Individual

Table 3 (Model 3) presents the within- and between-person predictors for the individual domain. Females reported lower rates of HV over time. Students from parents who had a college degree (compared with those who did not) reported, on average, higher rates of HV over time. At the within-person level, at time points when individuals reported higher rates of empathy compared with their “typical” levels, they, in turn, reported lower rates of HV at the same time point. Conversely, at time points when individuals reported higher rates of impulsivity and traditional masculinity compared with their “typical” levels, they, in turn, reported higher rates of HV at the same time point. At the between-person level, on average, individuals who reported higher average rates of empathy, impulsivity, and traditional masculinity reported higher rates of HV across time.

**Table 3.** Estimates of Fixed and Random Effects From a Series of Growth Models Predicting Homophobic Victimization.

Parameter estimates (*SE*)								
*Variables at each level*	Model 1	Model 2	Model 3	Model 4	Model 5	Model 6	Model 7	Model 8
Fixed effects								
Intercept	0.38*** (0.016)	0.44*** (0.038)	0.43*** (0.036)	0.49*** (0.038)	0.44*** (0.038)	0.46*** (0.038)	0.50*** (0.039)	0.47*** (0.037)
Linear slope	0.14*** (0.019)	0.14*** (0.019)	0.14*** (0.019)	0.15*** (0.019)	0.15*** (0.043)	0.15*** (0.019)	0.15*** (0.019)	0.15*** (0.019)
Quadratic slope	−0.04*** (0.006)	−0.04*** (0.006)	−0.04*** (0.006)	−0.04*** (0.006)	−0.04** (0.006)	−0.04*** (0.006)	−0.04*** (0.006)	−0.05*** (0.006)
Demographics								
Female		−0.18*** (0.027)	−0.13*** (0.027)	−0.21*** (0.027)	−0.17*** (0.028)	−0.17*** (0.027)	−0.17*** (0.027)	−0.19*** (0.027)
Parent education: College educated		0.10*** (0.031)	0.09*** (0.030)	0.09** (0.03)	0.11*** (0.032)	0.10*** (0.031)	0.12*** (0.031)	0.09** (0.03)
Parent education: Graduate school		0.03 (0.036)	0.02 (0.034)	0.03 (0.035)	0.04 (0.037)	0.05 (0.037)	0.07 (0.036)	0.02 (0.034)
Age		0.002 (0.013)	0.004 (0.013)	−0.01 (0.013)	0.02 (0.013)	0.001 (0.013)	−0.01 (0.013)	−0.01 (0.013)
African American		−0.05 (0.032)	−0.06* (0.032)	−0.11*** (0.031)	−0.06 (0.033)	−0.10** (0.033)	−0.20*** (0.038)	−0.10** (0.036)
Other		−0.03 (0.042)	−0.05 (0.040)	−0.05 (0.038)	−0.04 (0.042)	−0.06 (0.042)	−0.10** (0.041)	−0.06 (0.038)
Individual level								
WP empathy			−0.05*** (0.010)					−0.02* (0.010)
WP impulsivity			0.04*** (0.011)					0.01 (0.011)
WP dominance			0.02 (0.010)					0.01 (0.010)
WP traditional masculinity			0.07*** (0.017)					0.06*** (0.017)
BP empathy			0.05* (0.025)					0.06* (0.027)
BP impulsivity			0.19*** (0.021)					0.13*** (0.023)
BP dominance			0.02 (0.017)					−0.01 (0.018)
BP traditional masculinity			0.22*** (0.043)					0.15*** (0.041)
Family level								
WP family violence				0.09*** (0.010)				0.09*** (0.010)
WP parental monitoring				−0.04** (0.013)				−0.03* (0.013)
WP family support				0.01 (0.018)				0.08*** (0.02)
BP family violence				0.17*** (0.020)				0.11*** (0.021)
BP parental monitoring				−0.10*** (0.028)				−0.09** (0.029)
BP family support				0.02 (0.048)				0.03 (0.050)
Peer level								
WP peer support					−0.12*** (0.016)			−0.10*** (0.017)
BP peer support					−0.03 (0.036)			0.09* (0.04)
School level								
WP school belonging						−0.09*** (0.016)		−0.07*** (0.015)
WP adult support						−0.07*** (0.017)		−0.05** (0.018)
BP school belonging						−0.17*** (0.042)		−0.003 (0.039)
BP adult support						−0.03 (0.042)		−0.05 (0.047)
Neighborhood level								
WP neighborhood violence							0.06*** (0.012)	0.02 (0.012)
BP neighborhood violence							0.16*** (0.022)	0.03 (0.021)
Random effects								
Within-person intercept	0.18*** (0.012)	0.18*** (0.012)	0.17*** (0.012)	0.17*** (0.011)	0.17*** (0.011)	0.17*** (0.011)	0.18*** (0.012)	0.16*** (0.010)
Between-person intercept	0.23*** (0.029)	0.23*** (0.028)	0.21*** (0.027)	0.20*** (0.026)	0.23*** (0.028)	0.22*** (0.027)	0.22*** (0.027)	0.20*** (0.024)
Linear slope	0.14*** (0.044)	0.14*** (0.044)	0.15*** (0.044)	0.15*** (0.042)	0.15*** (0.043)	0.14*** (0.043)	0.14*** (0.043)	0.16*** (0.041)
Quadratic slope	0.01** (0.004)	0.01** (0.004)	0.01** (0.004)	0.01** (0.004)	0.01** (0.004)	0.01** (0.004)	0.01** (0.004)	0.01*** (0.004)
Fit indices								
−2 Log-likelihood	11,925.888	11,870.884	11,620.828	11,549.686	11,805.146	11,760.370	11,781.958	11,319.152
AIC	11,945.889	11,902.885	11,668.829	11,593.687	11,841.147	11,800.371	11,817.958	11,395.152
BIC	12,013.867	12,011.650	11,831.977	11,743.240	11,963.508	11,936.328	11,940.319	11,653.470
Degrees of freedom	10	16	24	22	18	20	18	38

*Note.* Male is the reference category for gender, and White is the reference category for race. Model 1 is a conditional growth model with linear and quadratic effects of growth that allows growth to vary randomly between people. Model 2 added demographic variables. Model 3 includes individual-level variables. Model 4 includes family-level variables. Model 5 includes peer-level variables. Model 6 includes school-level variables. Model 7 includes neighborhood-level variables. Model 8 includes variables across all social ecological domains. Intercept, random linear slope, and quadratic slope were allowed to co-vary. Covariances are not shown for ease of reading. WP = within-person; BP = between-person; SES = socioeconomic status; AIC = Akaike information criterion; BIC = Bayesian information criterion.

* *p* < .05. **p < .01. ***p < .001.

#### Family

Table 3 (Model 4) presents the within- and between-person predictors for the family domain. At the within-person level, at time points when individuals reported higher rates of family violence compared with their “typical” levels, they, in turn, reported higher rates of HV at the same time point. Conversely, at time points when individuals reported higher rates of parental monitoring compared with their “typical” levels, they, in turn, reported lower rates of HV at the same time point. At the between-person level, on average, individuals who reported higher average rates of family violence reported higher rates of HV across time. Individuals who reported higher average rates of parental monitoring reported lower rates of HV across time.

#### Peer

Table 3 (Model 5) presents the within- and between-person predictors for the peer domain. At the within-person level, at time points when individuals reported higher rates of peer support compared with their “typical” levels, they, in turn, reported lower rates of HV at the same time point.

#### School

Table 3 (Model 6) presents the within- and between-person predictors for the school domain. At the within-person level, at time points when individuals reported higher rates of school belonging and adult support compared with their “typical” levels, they, in turn, reported lower rates of HV at the same time point. At the between-person level, on average, individuals who reported higher average rates of school belonging reported lower rates of HV across time.

#### Neighborhood

Table 3 (Model 7) presents the within- and between-person predictors for the neighborhood domain. At the within-person level, at time points when individuals reported higher rates of neighborhood violence compared with their “typical” levels, they, in turn, reported higher rates of HV at the same time point. At the between-person level, on average, individuals who reported higher average rates of neighborhood violence reported higher rates of HV across time.

#### Competing model

Table 3 (Model 8) presents the within- and between-person predictors for the competing model. As a final step, we included predictors across all social domains in one competing model to examine key predictors while controlling for all other variables. At the within-person level, at time points when individuals reported higher rates of empathy, parental monitoring, peer support, school belonging, and adult support compared with their “typical” levels, they, in turn, reported lower rates of HV at the same time point. Conversely, at time points when individuals reported higher rates of traditional masculinity, family violence, and family support compared with their “typical” levels, they, in turn, reported higher rates of HV at the same time point. At the between-person level, on average, individuals who reported higher average rates of empathy, impulsivity, traditional masculinity, and family violence reported higher rates of HV across time. Individuals who reported higher average rates of parental monitoring reported lower rates of HV across time.

## Discussion

Homophobic name-calling and other forms of bias-based aggression are quite prevalent among youth. Often occurring among friends, this form of homophobic aggression during early adolescence is strongly inﬂuenced by peers and rooted in gender and masculinity (Birkett & Espelage, 2015). Over 90% of our sample identified as heterosexual, so it is important to note that sexual and gender minority students are significantly more likely to be victimized (Friedman et al., 2011). Homophobic name-calling victimization has been associated with low academic performance, depression, suicide, higher incidence of risky sexual behaviors, and substance abuse, among other adverse outcomes (Almeida et al., 2009; Espelage et al., 2008; Poteat & Espelage, 2007; Rivers, 2001). Adolescent males are also more likely to perpetrate homophobic aggression, especially when they support heteronormative norms (i.e., traditional masculinity) (Espelage et al., 2008). Thus, the present study contributes to the literature by identifying risk and protective factors within and across the social ecology that contribute to longitudinal patterns of homophobic name-calling perpetration and victimization among early adolescents.

The social-ecological factors which influence homophobic name-calling victimization and perpetration can be found beyond the individual domain, and include peers, family, the school, and the community. Bronfenbrenner (1979) proposed that attitudes and behaviors are shaped by external factors across different environments. Identifying the risk and protective factors for homophobic name-calling in different contexts can inform the development of prevention efforts that can be embedded in multiple areas of an adolescents’ life including their peer groups, schools, families, and communities. The results from this study are extensive given the depth and magnitude of the analyses. The between- and within-person relationships and the number of variables at different domains of the social ecology for homophobic name-calling involvement (victimization and perpetration) are best understood with a risk and protective factor framework. In addition, the discussion of the findings is focused on the relationships found when each predictor was entered into the model rather than the full model as the full model may mask the effect of some variables over others. However, findings from the competing models were discussed when results contradicted findings from the initial models. The strength of an association is bolstered when evidence was found at both levels (both within- and between-person) and also in the final model, signaling that these relationships may play a stronger role in predicting homophobic name-calling than associations only found at one level of analysis.

### Risk Factors

Within the individual domain, impulsivity had significant positive associations with homophobic name-calling perpetration and victimization at both within- and between-person levels of variability. These results suggest that impulsivity is a significant risk factor for homophobic name-calling involvement, a finding that is consistent with previous literature (Fanti & Kimonis, 2012; Merrin et al., 2018). Highly impulsive individuals may have difficulties with self-control and may be more reactive and prone to engage in bullying in general and homophobic name-calling perpetration in particular (Walters & Espelage, 2018). In addition, individuals with high levels of impulsivity may make comments or behave in ways without thinking that may put them in situations where bullying and homophobic name-calling victimization are more likely to occur (Fanti & Kimonis, 2012). Given the associations between impulsivity and homophobic name-calling perpetration and victimization, prevention programs should target impulsive behavior and cognition as a modifiable factor in the etiology of homophobic name-calling.

Similarly, social dominance was a risk factor for higher homophobic name-calling perpetration, but not victimization. Traditional masculinity was also significantly associated with higher homophobic name-calling perpetration only at the between-person level, and higher homophobic name-calling victimization at both the within- and between-person levels. These associations offer support for previous work, suggesting that students may use social dominance to assert their masculinity and are therefore more likely to be perpetrators (Pellegrini & Long, 2002; Poteat & DiGiovanni, 2010). In addition, it is possible that individuals who subscribe to a traditional masculine ideology may be associating with peers who also subscribe to this ideology, therefore increasing the likelihood of homophobic name-calling victimization, especially since the majority of homophobic name-calling victimization occurs among friends (Tucker, Ewing, Espelage, Green, & de lH Pollard, 2016). Early adolescents’ need to exert dominance over peers, especially among boys, could be redirected by prevention programs such as peer competition that use social dominance to encourage academic achievement or athletic performance rather than homophobic name-calling and aggression. In addition, others have called for adults to act as role models and promote educational efforts among early adolescents that decouple traditional masculinity, power, and authority from exerting dominance and homophobic name-calling (Birkett & Espelage, 2015).

Consistent with the literature, results indicated that family violence was a risk factor for higher homophobic name-calling perpetration and victimization both between person- and within-person (Espelage et al., 2000; Espelage et al., 2012; Voisin & Hong, 2012). These associations suggest that experiencing family violence in the home may play a dual role, encouraging the perpetration of further violence among peers and predisposing early adolescents to experiencing violence outside the home environment themselves. The dual roles of family violence in homophobic name-calling support the theories of Intergenerational Transmission of Violence and the concept of poly-victimization in which experiencing victimization early in life increases the risks for other forms of violence victimization (Espelage et al., 2012). Overall, these findings suggest that strategies to reduce and prevent homophobic name-calling involvement must address the normalization of violence in the home.

Contrary to our hypothesis, family social support emerged as a risk factor, with a significant positive association for homophobic name-calling perpetration at the between-person level, but not within-person. Similarly, a significant positive association between family social support and homophobic name-calling victimization emerged at the within-level but only in the competing model. These findings are inconsistent with the literature that has found that youth engaging in bullying perpetration or experiencing victimization typically have lower family support (Duggins, Kuperminc, Henrich, Smalls-Glover, & Perilla, 2016; Perren & Hornung, 2005). Findings may be due to the nature of bias-based aggression. Specifically, homophobic attitudes may be learned and upheld within a family, and therefore youth who have high support from homophobic family members may also be more likely to engage in homophobic name-calling perpetration. Future studies should measure bias-based attitudes among family members to further our understanding of the role family support has on homophobic name-calling perpetration among adolescents.

Finally, exposure to neighborhood violence was also a risk factor associated with higher levels of homophobic name-calling perpetration and victimization at both the within- and between-person levels. It is possible that students who live in neighborhoods with higher rates of violence also attend schools where homophobic name-calling is more likely to occur, and violence witnessed in these neighborhoods may translate to the adoption of views supportive of homophobic attitudes. For example, researchers have found bidirectional associations between school and neighborhood violence where both contexts influence each other (Mateu-Gelabert & Lune, 2003). However, some of these associations were not significant when all variables were considered in the model, suggesting that more immediate ecological variables (e.g., individual domain) may play a stronger role than neighborhood violence in predicting homophobic name-calling.

### Protective Factors

Within the individual domain, significant associations were found between higher empathy and lower homophobic name-calling perpetration at both the within-person and between-person levels. These results support previous literature underscoring empathy as a protective factor of homophobic name-calling perpetration (Poteat et al., 2012). Empathy may play a regulatory role, where students who can feel empathy for a victim may not engage in homophobic name-calling to the same degree as unempathetic peers due to perspective-taking or other mediating mechanisms (Poteat et al., 2012). Having higher empathy could also signal higher levels of social-emotional skills needed to handle conflict in social situations without resorting to homophobic name-calling. Empathy is a potentially malleable construct that could be a target of intervention programming aimed at reducing involvement in homophobic name-calling perpetration. For example, the prevention program Second Step which focused on increasing levels of empathy among middle school students had a positive impact on homophobic name-calling perpetration (Espelage, Low, Van Ryzin, & Polanin, 2015; Espelage, Van Ryzin, & Holt, 2018).

However, contradictory results emerged for empathy and homophobic name-calling victimization at the within- and between-person levels, where higher empathy within-person was associated with lower homophobic name-calling victimization but at the between-person level it was associated with higher homophobic name-calling victimization. It is possible that students who are perceived as more empathetic by their peers (between-person) may be more likely to be targets of homophobic name-calling due to negative perceptions of empathy as a weakness or feminine trait. Conversely, empathy may still be protective at times when individuals are experiencing higher empathy compared with their own typical levels due to yet unexplored mechanisms. In addition, our measure of empathy did not distinguish between the multiple dimensions of affective versus cognitive empathy which have been shown to function differently in traditional bullying and cyberbullying; therefore, further examination is needed to interpret the associations uncovered in the present study (Ang & Goh, 2010; Topcu & Erdur-Baker, 2012).

Within the family domain, results indicated that higher parental monitoring was protective and associated with lower homophobic name-calling perpetration and victimization at both the within- and between-person levels. The results are consistent with the literature, where adequate supervision from parents can buffer against bullying behaviors through peer selection (e.g., children with more involved parents tend to select fewer deviant peers, therefore are less likely to be victimized), and parents who monitor their children can actively reduce opportunities to engage in bullying behaviors such as homophobic name-calling (Low & Espelage, 2014).

When considering the peer domain, peer social support was examined as a protective factor for homophobic name-calling involvement. Significant negative associations were found between peer social support and homophobic name-calling perpetration and victimization at the within-person level. At times when individuals were experiencing higher levels of peer social support (compared with their typical levels), they were also less likely to engage in HP and experience victimization. Contradicting these relationships, the associations between peer social support and both homophobic name-calling perpetration and victimization were not significant at the between-person levels in the peer model, but they became significant and positive when all variables were entered in the competing model. Because homophobic name-calling is inherently social in nature, students who have more peers overall (higher social support when compared with others) may be more likely to engage in these behaviors due to having greater opportunities to interact with peers. However, at times when the student has greater social support than what they typically experience, this may still be protective. These findings illustrate the need to disentangle within- and between-person relationships when examining interpersonal factors such as social support from peers.

Within the school-domain, school belonging and social support from adults at school were examined as protective factors for homophobic name-calling perpetration and victimization. School belonging was protective for homophobic name-calling perpetration and victimization both within- and between-person levels of analysis. These results support a growing body of evidence suggesting a protective effect of school belonging on several aggressive behaviors (Espelage et al., 2019; Hong & Garbarino, 2012; Toomey et al., 2012). In addition, higher social support from adults at school was protective for homophobic name-calling perpetration at both within- and between-person levels of analysis, but was only significantly associated with lower homophobic name-calling victimization at the within-person level. Students who feel a greater sense of belonging at school and feel more connected to adults at school may be more likely to describe their school environment as positive, may also have better relationships with peers, and are therefore less likely to victimize others or be victimized by their peers.

### Limitations

Several limitations of this study should be noted. The sample for this study was collected from one U.S. Midwest city, which limits the generalizability of the findings. In addition, all measures were self-report, which could potentially yield biased results. However, all measures used in this study had a high degree of internal consistency and validity. Another limitation is that the study assessed sexual orientation only at Wave 1 because the school district received complaints about this question from parents and only 0.5% of the sample reported identifying as anything other than heterosexual. Finally, the sample used in this study was predominantly White and African American, which limits the generalizability of these findings to students of other races or ethnicities.

### Implications for Theory and Practice

Despite these limitations, the current study is a significant contribution to the literature on homophobic name-calling and bias-based aggression because it incorporates multiple domains of the social-ecological framework in a longitudinal design and includes both risk and protective factors while at the same time accounting for within- and between-person differences. The results illustrated a complex and comprehensive picture of the lives of youth who participate in these behaviors and provide potential avenues for intervention and further scientific inquiry. Schools with violence prevention efforts must look at the neighborhood domain and the family as key partners and allies for prevention while also paying attention to students’ social emotional competencies to address risk and protective factors at the individual domain. Specifically, prevention programs should target empathy, impulsivity, and aim to reduce the risk factors of social dominance and traditional masculinity through psycho-educational interventions. Another important area of application is increasing school belonging and social support from peers and adults as these domains emerged as protective factors for homophobic name-calling.

Due to the evidence in this article, the same construct manifested differently when accounting for intra-individual changes (within-person) rather than just differences between people. Therefore, we recommend future research to employ a similar approach to better understand how homophobic name-calling develops over time. Future research on homophobic name-calling should consider expanding on the gendered nature of this phenomenon by focusing on heteronormative gender roles that promote both homophobic name-calling perpetration and victimization. In addition, future studies should include transgender and other gender nonconforming individuals in their analysis as well as students at the intersections of gender, sexual identity, and race. These types of studies can further our understanding of homophobic name-calling by capturing how students negotiate their marginalized identities in different contexts and how heterosexism, racism, and transphobia influence bias-based aggression, both in-person and online.

## Supplemental Material

sj-docx-1-jea-10.1177_02724316211002271 – Supplemental material for Social-Ecological Predictors of Homophobic Name-Calling Perpetration and Victimization Among Early Adolescents Click here for additional data file. Supplemental material, sj-docx-1-jea-10.1177_02724316211002271 for Social-Ecological Predictors of Homophobic Name-Calling Perpetration and Victimization Among Early Adolescents by Alberto Valido, Gabriel J. Merrin, Dorothy L. Espelage, Luz E. Robinson, Kyle Nickodem, Katherine M. Ingram, America J. El Sheikh, Cagil Torgal and Javari Fairclough in The Journal of Early Adolescence

sj-xlsx-2-jea-10.1177_02724316211002271 – Supplemental material for Social-Ecological Predictors of Homophobic Name-Calling Perpetration and Victimization Among Early Adolescents Click here for additional data file. Supplemental material, sj-xlsx-2-jea-10.1177_02724316211002271 for Social-Ecological Predictors of Homophobic Name-Calling Perpetration and Victimization Among Early Adolescents by Alberto Valido, Gabriel J. Merrin, Dorothy L. Espelage, Luz E. Robinson, Kyle Nickodem, Katherine M. Ingram, America J. El Sheikh, Cagil Torgal and Javari Fairclough in The Journal of Early Adolescence
