# Supplementary material for: Social-Ecological Predictors of Homophobic Name-Calling Perpetration and Victimization Among Early Adolescents
Source: J Early Adolesc. 2021 Apr 29;42(9):1115–51. doi: 10.1177/02724316211002271 (PMC9623402; doi:10.1177/02724316211002271)
Supplement: sj-docx-1-jea-10.1177_02724316211002271 – Supplemental material for Social-Ecological Predictors of Homophobic Name-Calling Perpetration and Victimization Among Early Adolescents [file sj-docx-1-jea-10.1177_02724316211002271.docx]

Supplemental Table 1. Socio-ecological Measures Description.

| **Measure Description** | **Internal Consistency Reliability Omega (ω)** | | | |
| --- | --- | --- | --- | --- |
| ***Outcomes*** | **W1** | **W2** | **W3** | **W4** |
| *Homophobic Name-calling Perpetration* was assessed using the first 5 items from the 10-item Homophobic Content Agent Target (HCAT) scale (Poteat & Espelage, 2005, 2007). Students were asked “How many times in the last 30 days did YOU say homo, gay, lesbo, or fag to the following individuals?” Then they were presented the five items: (1) a friend, (2) someone you did not know well, (3) someone you did not like, (4) someone you thought was gay or lesbian, and (5) someone you did not think was gay or lesbian. Response options were0 (*Never*), 1 (*1 or 2 times*), 2 (*3 or 4 times*), 3 (*5 or 6 times*), and 4 (*7 or more times*). | .90 | .88 | .89 | .88 |
| *Homophobic Name-calling Victimization* was measured with the second 5 items from the HCAT. the victimization scale consisted of the same items and response options as the perpetration scale, except students were asked how often others (e.g., friends) called them homophobic epithets. Validity: Exploratory factor analysis supports the 10-item HCAT as a two-factor (perpetration and victimization) instrument (Poteat & Espelage, 2005, 2007). The perpetration scale has been positively correlated with bullying for both males and females and negatively correlated with empathy. Likewise, the victimization scale positively correlates with measures of bullying victimization . | .86 | .89 | .88 | .86 |
| ***Individual Level*** |  |  |  |  |
| *Impulsivity.* A 4-item scale developed by Bosworth and colleagues (1999) asked students to indicate how often they would say the following statements about themselves: (1) “I have a hard time sitting still,” (2) “I start things but have a hard time finishing them,” (3) “I do things without thinking,” and (4) “I need to use a lot of self-control to keep out of trouble.” Response options were 0 (*Never*), 1 (*Seldom*), 2 (*Sometimes*), 3 (*Often*), and 4 (*Always*). Validity: In multiple samples the scale has shown adequate reliability (α > .60) as well as convergent validity with positive associations with bullying (Espelage et al., 2001) and delinquency (Low & Espelage, 2014) | .79 | .76 | .79 | .81 |
| *Empathy* was measured with the 5-item Empathy subscale of the Teen Conflict Survey (Bosworth & Espelage, 1995). The items assessed both cognitive (“*I can listen to others*;” “*Kids I don’t like can have good ideas*;” “*I trust people who are not my friends*”) and affective (“*I get upset when my friends are sad*;” “*I am sensitive to other people’s feelings, even if they are not my friends*”) empathy. Response options for each item were 0 (*Never*), 1 (*Seldom*), 2 (*Sometimes*), 3 (*Often*), and 4 (*Always*). Validity: Previous studies with heterogeneous samples found adequate reliability (α > .60) as well as convergent validity with a positive association with prosocial behavior (McMahon et al., 2006) and discriminant validity with a negative association positive attitudes toward bullying (Walters & Espelage, 2018). | .77 | .72 | .76 | .80 |
| *Traditional Masculinity* was assessed with the 7-item traditional masculinity subscale of the Adolescent Masculinity Ideology in Relationships Scale (AMIRS; Chu, Porche, & Tolman, 2005). Students were asked how much they agree with statements such as, “It’s important for a boy to act like nothing is wrong, even when something is bothering him," and “In a good dating relationship the boy gets his way most of the time." Response options were0 (*Strongly Disagree*), 1 (*Disagree*), 2 (*Agree*), and 3 (*Strongly Agree*). Validity: Scale items were developed through ethnographic observations and semistructured interviews with adolescent boys (see Chu, Porche, & Tolman, 2005 for full details). A pilot study led to scale refinement. An EFA found the AMIRS to be a unidimensional measure, with responses negatively correlated with the Attitudes towards Women Scale for Adolescents, which is a measure of unconventional attitudes toward women’s roles and rights (Galambos et al., 1985) , providing evidence of discriminant validity. | .81 | .85 | .86 | .83 |
| *Social Dominance.* Drawing on the literature on social information processing related to proactive aggression (Dodge & Coie, 1987) and social dominance theory, we adapted a 6-item teacher checklist on youth’s need for dominance among peers and their use of aggression to get their way (Pellegrini & Long, 2002). Students were asked how well each item described them, such as “I enjoy being the center of attention,” “I am usually a leader of my group of friends,” and “I can force others to do what I want.” Response options were 0 (*Does not describe me at all*) through 4 (*Describes me well*). Validity: As a checklist, teacher report reliability was .88 across middle school grades and demonstrated convergent validity with bullying (Pellegrini & Long, 2002). | .85 | .83 | .85 | .87 |
| ***Family Level*** |  |  |  |  |
| *Family conflict and hostility* was measured with the 3-item Family Conflict and Hostility Scale from the Rochester Youth Development Study (Thornberry et al. 2003). Students were asked (1) “How often is there yelling, quarreling, or arguing in your household?”, (2) “How often do family members lose their temper or blow up for no good reason?" and (3) "How often are there physical fights in the household, like people hitting, shoving, or throwing things?” Response options were0 (*Never*), 1 (*Seldom*), 2 (*Often*), 3 (*Always*). Validity: As part of a larger longitudinal measurement model significantly linking family conflict to later crime and arrest as an adult, the items had factor loadings of .92, .81, and .76, respectively, with model fit of CFI = .97 and RMSEA = .03 (Krohn et al., 2011) | .80 | .83 | .83 | .81 |
| *Parental Monitoring* was assessed with eight items from the Parental Supervision subscale from the Seattle Social Development Project (Arthur et al., 2002; Low & Espelage, 2014). The scale measures perceptions of established familial rules and perceived parental awareness regarding schoolwork and attendance, peer relationships, alcohol or drug use, and weapon possession. Students were asked items such as: “*My family has clear rules about alcohol and drug use.*” and “*My parents ask if I’ve gotten my homework done.*” Response options were 0 (*Never)*, 1 (*Rarely*), 2 (*Sometimes)*, and 3 (*Often*). Validity: In a sample of over 10,000 middle and high school students, internal consistency reliability was > .69 across grades and gender and was inversely related to drug and alcohol use (Arthur et al., 2002). | .90 | .91 | .91 | .92 |
| *Family Social Support* was measured with a 3-item family subscale from the Vaux Social Support Record (VSSR; Vaux, 1988). Students were asked to indicate how many people in their life fit the following descriptions: “There are people in my family…” (1) “I can talk to, who care about my feelings and what happens to me,” (2) “I can talk to, who give me good suggestions and advice about my problems,” and (3) “who help me with practical problems, like helping me get somewhere or helping me with a project.” Response options were0 (*None*), 1 (*Some*), and 2 (*A lot*). Validity: The VSSR was developed with 10 samples representing a variety of ages and backgrounds (Vaux et al., 1986). Over a 6-week period, test-retest reliabilities across the 10 samples ranged from .71 - 80. The measure also demonstrated convergent validity with positive correlations with other support measures and discriminant validity with negative correlations with measures of loneliness. | .83 | .79 | .85 | .83 |
| ***Peer Level*** |  |  |  |  |
| *Peer Social Support* was measured with a 3-item subscale from the VSSR (Vaux, 1988). Students were asked to indicate how many people in their life fit the following descriptions: “I have friends …” (1) “I can talk to, who care about my feelings and what happens to me,” (2) “I can talk to, who give me good suggestions and advice about my problems,” and (3) “who help me with practical problems, like how to get somewhere.” Response options were 0 (*None*), 1 (*Some*), and 2 (*A lot*). Validity: see Vaux et al (1986) for details and the summary under *Family Social Support*. | .87 | .84 | .85 | .84 |
| ***School and Neighborhood Level*** |  |  |  |  |
| *School belonging* was measured with 4 of the 18 items from the Psychological Sense of School Members Scale (Goodenow, 1993). Students were asked how much they agree with the statements: (1) "I feel proud of belonging to this school," (2) "I am treated with as much respect as other students," (3) "The teachers here respect me," and (4) "There is at least one teacher or other adult in this school I can talk to if I have a problem." Response options were0 (*Strongly Disagree*), 1 (*Disagree*), 2 (*Agree*), and 3 (*Strongly Agree*). Validity: The original set of items were iteratively developed through a series of pilot studies with a diverse sample of middle and junior high school students (Goodenow, 1993). However, the negatively worded items have been problematic (Hagborg, 1994; Ye & Wallace, 2014). The 4 items used here have been negatively correlated with aggression victimization (Espelage & Holt 2001; Poteat & Espelage 2005), which provides evidence of discriminant validity. | .66 | .73 | .74 | .76 |
| *Social Support from Adults at School* was measured with a 3-item subscale from the VSSR (Vaux, 1988). Students were asked to indicate how many people in their life fit the following descriptions: “At school, there are adults …” (1) “I can talk to, who care about my feelings and what happens to me,” (2) “I can talk to, who give me good suggestions and advice about my problems,” and (3) “who help me with practical problems, like helping me get somewhere or help me with a project.” Response options were 0 (*None*), 1 (*Some*), and 2 (*A lot*). Validity: see Vaux et al (1986) for details and the summary under *Family Social Support*. | .80 | .81 | .84 | .84 |
| *Exposure to community violence* was measured with five items from the 12-item Children’s Exposure to Community Violence scale (Low & Espelage, 2014; Richters & Martinez, 1990). Students are asked, “How often do you hear or see the following in your neighborhood, school, or at your home?”: (1) “I have heard guns being shot”, (2) “I have seen somebody arrested”, (3) “I have seen drug deals”, (4) “I have seen somebody being beaten up”, and (5) “I have seen gangs." Response options were 0 (*Never)*, 1 (*Rarely*), 2 (*Sometimes)*, and 3 (*Often*). Validity: In Low & Espelage (2014), internal consistency reliability was .91 with convergent validity evidenced by positive associations with delinquency, fighting, and bullying. | .92 | .92 | .92 | .92 |
